# Enhancing Species Distribution Models by Considering Dispersal Ability

**DOI:** 10.1002/ece3.73425

**Published:** 2026-04-13

**Authors:** Mengge Duan, Jiahua Xing, Xiudeng Zheng, Chaodong Zhu, Huijie Qiao

**Affiliations:** ^1^ State Key Laboratory of Animal Biodiversity Conservation and Integrated Pest Management, Institute of Zoology, Chinese Academy of Sciences Beijing China; ^2^ College of Life Science, University of Chinese Academy of Science Beijing China; ^3^ Lab of Animal Behavior and Conservation, School of Life Sciences Nanjing University Nanjing China

**Keywords:** climate change, dispersal capability, dispersal constraint, habitat projection, species distribution model, underestimate risk

## Abstract

Climate change is driving shifts in species' geographic ranges, and understanding these changes is critical for effective biodiversity conservation. Species Distribution Models (SDMs) are widely used to predict the potential distribution of a given species under climate change. However, most studies focus solely on shifts in suitable habitats without considering whether species can reach these areas within a relevant timeframe. This omission often results in overestimation of future distributions and underestimation of extinction risk. Here, we designed an experiment to assess the impact of dispersal constraints on SDM predictions using 10 species with varying dispersal abilities. We compared the potential future distribution of species under two scenarios: the first, incorporating their dispersal ability and the second, assuming unlimited dispersal. Our results demonstrate that ignoring dispersal leads to an overprediction of suitable habitats for species, which may result in underestimating species extinction risk or overestimating the risk of range expansions by invasive species. This underscores the critical need to integrate dispersal dynamics into SDMs to enhance the accuracy of biodiversity projections. By incorporating realistic movement constraints, SDMs can provide more reliable predictions, leading to improved conservation planning, better management of invasive species, and more effective biodiversity conservation efforts under climate change.

## Graphical abstract

We designed an experiment using 10 species with varying dispersal abilities, comparing their potential future distributions under realistic dispersal constraints versus assumptions of unlimited dispersal. Our results demonstrate that ignoring dispersal limitations leads to a significant overprediction of suitable habitats. We highlight that this omission risks underestimating extinction probabilities for vulnerable species while overestimating the expansion potential of invasive species.

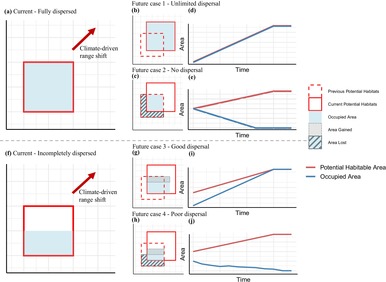

## 1 Introduction

Climate change is driving shifts in species' geographical ranges, and understanding these changes is essential for effective biodiversity conservation (Pecl et al. 2017). To anticipate how species may respond to future environmental conditions, Species Distribution Models (SDMs) are indispensable as they relate occurrences to a suite of environmental predictors to estimate the environmental conditions that constrain or facilitate species' presence (Elith and Leathwick 2009, Guisan and Thuiller 2005). However, dispersal ability is a crucial factor limiting the capacity of species to adapt to a changing world (Barber‐O'Malley et al. 2022). Traditional SDM applications assume species can immediately occupy all newly suitable habitats. Yet, this approach neglects the dispersal limitations of species (Bateman et al. 2013) and leads to overestimation of future distributions and invasion probability, and underestimation of extinction risk, potentially misleading conservation and invasive species management efforts.

SDMs are frequently parameterized using two dispersal assumptions: no‐dispersal versus full dispersal (Buse and Griebeler 2011, Peyre et al. 2020). However, in reality, dispersal rates are highly variable among species and governed by complex constraints. While some species exhibit rapid dispersal that allows them to quickly colonize new suitable habitats, many others have limited movement capabilities due to biological, ecological, or geographical barriers (Russell et al. 2015). For example, organisms such as amphibians and freshwater species are often restricted by specific microhabitats or watershed boundaries, whereas the dispersal of invasive species is influenced by traits like flight ability, environmental variables such as wind currents (Wilson et al. 2009; Zhang et al. 2023), and anthropogenic factors including agricultural trade (Fenn‐Moltu et al. 2022; Gippet et al. 2019). Consequently, relying on a full‐dispersal assumption often fails to capture these limitations, as many species are unable to disperse rapidly enough to track shifting climates (Russell et al. 2015). This leads to an overestimation of future ranges and a dangerous underestimation of extinction risks; a species may lose its current habitat due to climate change but fail to reach newly suitable areas, leading to local or global extinction (Bellard et al. 2012). Thus, models that ignore these dynamics may falsely indicate species persistence, misleading conservation and management efforts.

Similarly, overestimating potential distributions can also lead to an overestimation of invasion risk of invasive species. Many invasive species have dispersal constraints that limit their spread despite the presence of suitable climatic conditions in new regions. Traditional SDMs that do not account for dispersal ability may predict an overly rapid and widespread invasion, leading to unnecessary concern and misallocated management resources (Václavík and Meentemeyer 2009). In reality, an invasive species may take much longer to establish in a new area due to physical barriers, ecological resistance, or slow natural dispersal rates (Lázaro‐Lobo et al. 2025). Thus, failing to incorporate dispersal dynamics in SDMs can lead to unrealistic projections that may mislead conservation efforts and policy decisions.

Furthermore, the rate of dispersal is variable among species. While some species exhibit rapid dispersal rates that allow them to quickly colonize new habitats, others have highly limited movement capabilities due to biological, ecological, or geographical barriers (Russell et al. 2015). For example, some amphibians and reptiles have low dispersal rates due to their reliance on specific microhabitats, while freshwater species may be constrained by watershed boundaries. The dispersal ability of invasive species is influenced by species traits (flight ability), environmental variables (wind currents; Wilson et al. 2009, Zhang et al. 2023), and anthropogenic factors (agricultural trade, transportation of infested plants; Fenn‐Moltu et al. 2022, Gippet et al. 2019). Thus, without explicitly incorporating dispersal limitations into SDMs, projections of future invasive species distributions may not align with observed invasion patterns.

The limitations of traditional SDMs in accounting for dispersal dynamics have been widely recognized in ecological modeling (Allouche et al. 2008; Bateman et al. 2013; Cardador et al. 2013). Some studies have attempted to integrate dispersal models into SDMs by incorporating mechanistic dispersal simulations (Louvrier et al. 2020), metapopulation dynamics (Naujokaitis‐Lewis et al. 2013), or species‐specific movement ecology (Medrzycki et al. 2017). However, many SDM applications still rely on static correlative approaches that do not explicitly model how species move through landscapes over time. This represents a major gap in our ability to accurately predict biodiversity responses to global change.

**FIGURE 1:**
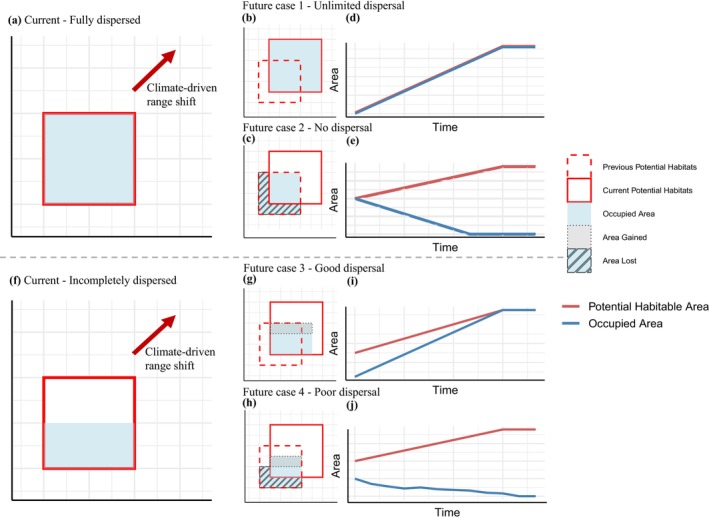
Conceptual framework of species distribution area dynamics under climate‐driven dispersal scenarios. The red arrows in panels a and f indicate shifts in species' potential suitable habitats driven by climate change. The upper panel illustrates scenarios where a species has fully colonized its potential distribution range (a). As climate change induces distribution range shifts, the species' dispersal ability dictates the spatial change with pre‐existing distributions (b, c), shaping the respective area change trajectories over time (d, e). The lower panel depicts scenarios where a species has not completely colonized its potential distribution range (f), leading to differential patterns of range expansion and overlap under future climatic shifts (g, h), with corresponding area dynamics over time (i, j). Red outlines denote initial range extents, while dashed red lines and shaded regions indicate newly gained or overlapping areas. In (a–c), (f–h), red solid boxes denote the species' potential distribution range, while dashed red boxes represent its previous potential distribution range. Blue‐shaded areas indicate regions currently occupied by the species. Gray areas with dotted boxes represent area gain, whereas striped areas denote area loss compared to the previous time segment.

In this study, we aim to bridge this gap by demonstrating how dispersal constraints influence SDM projections, using 10 species with different dispersal abilities as a case study. We hypothesized that species' inherent dispersal capacities determine both the magnitude and direction of biases in SDM projections. Weakly dispersing species should show the largest mismatch between potential and realized future ranges, whereas highly dispersing species, including invasive species, should exhibit the opposite pattern (Figure 1). By comparing future distribution projections under two scenarios: one assuming unlimited dispersal and one incorporating species‐specific dispersal limitations, we highlight the risks of overestimating both extinction and invasion threats when dispersal is not considered. Our findings emphasize the importance of incorporating dispersal dynamics into SDMs to improve their reliability for biodiversity conservation, pest management, and climate change adaptation planning.

## 2 Materials and Methods

### 2.1 Study Species, Study Area, and Climate Change Scenarios

The study examines the dynamic changes in the future distribution area of 10 species of birds, amphibians and insects: Sikkim treecreeper ( *Certhia discolor* ), Brown‐throated fulvetta ( *Fulvetta ludlowi* ), Black‐crowned scimitar babbler ( *Pomatorhinus ferruginosus* ), Striated laughingthrush (*Grammatoptila striata*), Large woodshrike ( *Tephrodornis virgatus* ), Common Chinese tree frog ( *Hyla chinensis* ), Plateau brown frog ( *Rana kukunoris* ), Codling moth (*Cydia pomonella*), South American tomato leafminer (*Tuta absoluta*), Colorado potato beetle ( *Leptinotarsa decemlineata* ), which encompass a diverse taxonomic range. These species include both endangered and protected species, as well as invasive species (Table S1) that are of growing concern for ecosystem stability. These species also exhibit a wide spectrum of dispersal ability (Table S1), ranging from the species with the poorest dispersal ability (Black‐crowned scimitar babbler) to the one with the best (Colorado potato beetle). This range of dispersal abilities makes our focal species well‐suited for testing our hypothesis.

The study area encompasses China, the third‐largest country in the world by area, renowned for its rich and diverse ecological types. Its vast territory spans a broad range of climate zones, from the cold temperate regions in the north to the tropical areas in the south (Mi et al. 2021). Additionally, the country features a variety of geographical environments, including expansive mountains, plateaus, plains, and coastal regions. These diverse landscapes contribute to China's status as one of the most biodiverse nations globally (Wang et al. 2020), with a notably high proportion of endemic species (Liu et al. 2003). This remarkable biodiversity is supported by the unique climatic and geographical conditions found across China, which create a variety of habitats for numerous species. Climate change, including rising temperatures, shifting precipitation patterns, and frequent extreme weather events, may significantly alter the survival conditions and distribution patterns of species, thereby affecting the structure and function of ecosystems.

The research focuses on examining the potential impacts of climate change on the habitat suitability and geographical distribution of species under two Representative Concentration Pathways (RCP4.5 and RCP8.5) scenarios (Pachauri et al. 2014). RCP4.5 represents a moderate emissions scenario, where global greenhouse gas emissions stabilize by mid‐century, while RCP8.5 reflects a high‐emissions pathway with continued increases in greenhouse gases, leading to significant warming (Pachauri et al. 2014). The study aims to model how these climate scenarios will influence the habitats of various species, particularly in areas where climate‐induced shifts are expected to occur. Given China's vast geographical and climatic diversity, the impacts of climate change on habitat suitability may vary significantly across different species.

### 2.2 Data Collection and Model Development

#### 2.2.1 Definition of Species Dispersal Capacity

Species' dispersal capacities were defined based on taxonomic group characteristics and available literature (Cayuela et al. 2020; Esch et al. 2021; Ferracini et al. 2012; Liu et al. 2012). For three different taxonomic groups, species dispersal ability is defined within a range of 0–50 km per year. Due to their limited mobility, the two amphibian species were assigned a dispersal ability of 1 km per year (Cayuela et al. 2020). The three invasive insect species have the highest reproductive capacity and potential for long‐distance dispersal through natural factors such as wind, and were assigned dispersal ability of 10–50 km per year (Chen and Dorn 2010, Esch et al. 2021, Ferracini et al. 2012, Liu et al. 2012; Table S1). The dispersal ability of the five bird species is estimated based on predicted natal dispersal modeling (Table S1), ranging from 0.5 to 14 km per year (Table S1).

Among the 10 species, four have dispersal abilities exceeding 10 km/year, including three invasive insects ( *L. decemlineata* , *T. absoluta*, and *C. pomonella*) and one resident bird ( *T. virgatus* ). Two species have dispersal abilities ranging from 2 km/year to 10 km/year, all of which are resident birds ( *F. ludlowi* and *C. discolor* ). The dispersal abilities of four species are below 2 km/year, including two amphibians ( *H. chinensis* and *R. kukunoris* ) and two resident birds ( *G. striata* and *P. ferruginosus* ).

#### 2.2.2 Species Occurrence Data

We obtained species occurrence records (GBIF.org 2025a, 2025b, 2025c, 2025d, 2025e, 2025f, 2025g, 2025h, 2025i, 2025j) from Global Biodiversity Information Facility (GBIF) databases, and references for *Cydia pomonella* (Yu 2025), *Tuta absoluta* (Xue et al. 2025), and *Leptinotarsa decemlineata* (Gao et al. 2022). To reduce sampling bias, occurrence records were preprocessed using the following steps. First, records without spatially explicit information were removed. Second, we retained only records with a coordinate uncertainty of ≤ 10 km, or those with missing uncertainty values. Third, to ensure data reliability, only records classified as HUMAN_OBSERVATION, OBSERVATION, PRESERVED_SPECIMEN, or MACHINE_OBSERVATION were included, and only records collected between 2000 and 2024 were considered. Finally, to match the spatial resolution of environmental layers and minimize spatial autocorrelation, only one occurrence per 0.1° × 0.1° grid cell (≈10 km) was retained (Moulatlet et al. 2025). All species occurrence data after processing are shown in Figure S1. Apart from the three invasive insects, all other species are native (Table S1). The number of occurrence records was 205 for *H. chinensis* , 21 for *R. kukunoris* , 122 for *P. ferruginosus* , 646 for *G. striata* , 59 for *F. ludlowi* , 202 for *C. discolor* , 1416 for *T. virgatus* , 5309 for *C. pomonella*, 575 for *T. absoluta*, and 9068 for *L. decemlineata* .

#### 2.2.3 Environmental Data

Key environmental predictors included variables related to temperature and precipitation. For invasive insect species, elevation was also incorporated as a bound variable, as these species are generally unable to establish populations above 3000 m (Willett et al. 2009). Species occurrence data consist of detailed species distribution points recorded in latitude and longitude. We used the same 19 climatic variables as WorldClim. These environmental data, with a resolution of 0.1° × 0.1°, were obtained from the Copernicus Climate Change Service (C3S) Climate Data Store (CDS; Wouters et al. 2021), which provides annual environmental data from 2000 to 2100. This database includes multiple climate models and different climate scenarios representing increasing greenhouse gas emissions. Based on model applicability, the GFDL‐ESM2M (NOAA) model was selected, along with the RCP4.5 and RCP8.5 climate scenarios. The current climate data were calculated based on climate data from 2000 to 2024 under the GFDL‐ESM2M (NOAA) model and RCP4.5 climate scenario. Future climate data were derived from annual projections (2025–2100) of the GFDL‐ESM2M (NOAA) under RCP4.5 and RCP8.5, with interannual variation reflecting changes in variable values (Figure S2). Elevation data, serving as a constraint factor, were obtained from the SRTM (https://www.earthdata.nasa.gov/data/instruments/srtm) database. To avoid multicollinearity, Pearson correlation analysis of these climate factors was performed using the ‘*cor*’ function from the ‘*Hmisc*’ package (Harrell and Harrell 2019). Variables were selected based on their correlation results (|r| < 0.7) and the biological characteristics of the species. Also, the variance inflation factors (VIFs) of the predictor variables for all species were below 10. The number of bioclimatic variables selected for modeling varied among species, ranging from three to four (Table S2). BIO01 (annual mean temperature) and BIO12 (annual precipitation) were the most frequently retained, indicating their central role in shaping species distributions. Temperature‐related variables (e.g., BIO02, BIO03, BIO04) and precipitation‐related variables (e.g., BIO15, BIO17, BIO19) were included in multiple species, reflecting the importance of both thermal conditions and moisture availability. These results suggest that species‐specific responses to temperature and precipitation govern habitat suitability, while the relative influence of individual variables differs among taxa. The correlations between the selected climate variables were low (|r| < 0.7, Ermokhina et al. 2023), avoiding multicollinearity.

#### 2.2.4 Species Distribution Modeling (SDMs)

We built species distribution models (SDMs) using the MaxEnt algorithm for each of the 10 focal species (Phillips and Dudík 2008). The number of background points was set as 1% of the number of valid grids in the study area multiplied by the number of environmental variables, and the points were randomly sampled. For the three invasive species and *T. virgatus* , global occurrence data were used, necessitating the use of global climate data. For other protected species, occurrence data were distributed across China and Southeast Asia, with selected climate data covering the range (longitude: 73°–136°, latitude: 3°–54°). These models projected future conditions in China. Prior to model construction, optimal MaxEnt parameter settings were identified using the corrected Akaike Information Criterion (ΔAICc) implemented in the ‘*ENMeval*’ R package (Kass et al. 2021). The ‘*ENMevaluate*’ function was employed to select the best combinations of feature classes (FC) and regularization multipliers (RM) based on block validation. Feature classes tested included linear (L), quadratic (Q), and product (P) features, as well as their combinations: L, Q, P, LQ, LP, QP, and LQP. The regularization multiplier (RM) was tested in the range of 1 to 3, with a step size of 0.5.

#### 2.2.5 Threshold Determination

After constructing the model with the optimal parameters, the True Skill Statistic (TSS) was used to determine the optimal binary threshold. This threshold was then applied to identify potentially suitable habitats for the species under both current and future environmental conditions.

#### 2.2.6 Dispersal Scenarios

To assess how species might occupy future suitable habitats, two dispersal scenarios were simulated (Figure 1):

#### 2.2.7 Unlimited Dispersal

Assumes species can colonize all newly suitable habitats instantaneously. There are no restrictions on dispersal speed, allowing species to spread to any region. As long as the climatic conditions meet the species' ecological niche for survival, the region can be considered a potential distribution area. In this scenario, the species' potential habitat suitability directly corresponds to its potential distribution range.

#### 2.2.8 Limited Dispersal

Incorporates species‐specific dispersal rates, estimated from movement velocities, to constrain potential future distributions. Species have a defined dispersal ability, meaning they can only establish in areas they can physically reach, provided that the local climate is suitable for their survival. In this scenario, the potential distribution area is determined by both the species' dispersal capacity and the suitability of the habitat.

Figure 1 illustrates the framework of two dispersal scenarios where the species has fully colonized its potential range or has not yet fully occupied its initial potential distribution, allowing for either a direct transition as climate‐induced shifts occur or differential range expansion patterns under future climate conditions (Figure 1a,f). For species fully dispersed, unlimited dispersal leads to a rapid filling of suitable habitat range (Figure 1b), showing a high synchronization between the potential distribution area and occupied area (Figure 1d), whereas no dispersal prevents it from filling new habitats (Figure 1c), which is reflected in the rapid decline of occupied area (Figure 1e). For species that haven't completely dispersed to all their suitable range, the effect of dispersal constraints becomes more pronounced. Under unlimited dispersal or strong dispersal scenarios, newly suitable habitats are rapidly colonized as climate change shifts the species' ecological niche (Figure 1g) and will eventually occupy all suitable areas. This leads to a steady increase in the occupied area, closely mirroring the expansion of the potential distribution area over time (Figure 1i). Under the limited or poor dispersal scenario, the species' ability to occupy newly suitable areas is restricted by its dispersal capacity (Figure 1h), which results in a slower and more fragmented range expansion, as only regions within the species' annual dispersal capacity can be colonized. As a consequence, the occupied area increases at a much slower rate compared to the unlimited dispersal scenario, creating a persistent gap between the potential and realized distribution (Figure 1j).

### 2.3 Model Evaluation and Analysis

#### 2.3.1 Model Evaluation

The performance of the species distribution models was assessed using the partial AUC (Cobos et al. 2019), TSS, and Cohen's Kappa (KAPPA) values.

#### 2.3.2 Projection of Potential Suitable Habitats

The species distribution model was projected onto China to predict the species' potential suitable habitat under current climatic conditions and future climate scenarios (RCP4.5 and RCP8.5) from 2025 to 2100. To minimize potential spatial bias caused by unequal grid cell sizes, the model was reprojected using the Eckert IV equal‐area projection (Budic et al. 2016).

#### 2.3.3 Dispersal Analysis

To evaluate the effect of dispersal limitations on SDM predictions, we compared the spatial extent and geographic changes under two dispersal scenarios (Section 2.2.1). Under the consideration of dispersal limitation, the maximum dispersal distance used for the species is shown in Table S1. The dispersal method was that each occurrence point was assigned four dispersal directions, and the dispersal distance along each direction was randomly calculated based on the maximum dispersal distance and an exponential distribution pattern, forming a buffer representing the dispersal result for that point. To more accurately assess the effects of dispersal on species with different dispersal capacities, the grid resolution was adjusted when a species' dispersal ability was smaller than the default grid size (11074.49 × 11074.49 m). Specifically, to avoid the influence of baseline raster size on species dispersal patterns, if a species' maximum annual dispersal distance was smaller than the default grid resolution (with a temporal interval of 1 year in the climate data), the grid resolution was set to match the species' maximum annual dispersal distance. Under an unlimited dispersal scenario, all potential suitable habitats were considered as potential distribution areas. Under a limited dispersal scenario, the current potential distribution was determined based on the species' maximum dispersal capacity. For native species, the current potential distribution was constrained by their maximum dispersal ability. For the invasive species, since the invasion period is relatively short, the current potential distribution was defined as 10 times the maximum dispersal distance buffer around known occurrence points. Annual potential distributions were estimated by combining potentially suitable areas with dispersal buffers. Potential suitable areas for each year were predicted using climate data and the MaxEnt model. Dispersal buffers were then iteratively calculated based on species' dispersal distance, dispersal angles, dispersal frequency (set to 1 for all species), and the potential distribution from the previous year. The dispersal simulation started from 2025, expanding from the current potential distribution. Calculate the potential distribution under two dispersal scenarios for the future (2025–2100) based on different climate scenarios (RCP4.5, RCP8.5).

## 3 Results

### 3.1 Model Evaluation

As a result, the TSS values for the models of all 10 species exceed 0.64, the Kappa values are all greater than 0.47, and the partial AUC values are above 1.59. These performance metrics collectively indicate the reliability and robustness of the models. Detailed model evaluation results for each species' MaxEnt model, including performance metrics and other relevant parameters, are presented in Table S2. These results indicate that the models for all species effectively capture species' distribution patterns, yielding high prediction accuracy and consistency across taxa.

### 3.2 Species Distribution Area Change Under Future Climate Scenarios

**FIGURE 2:**
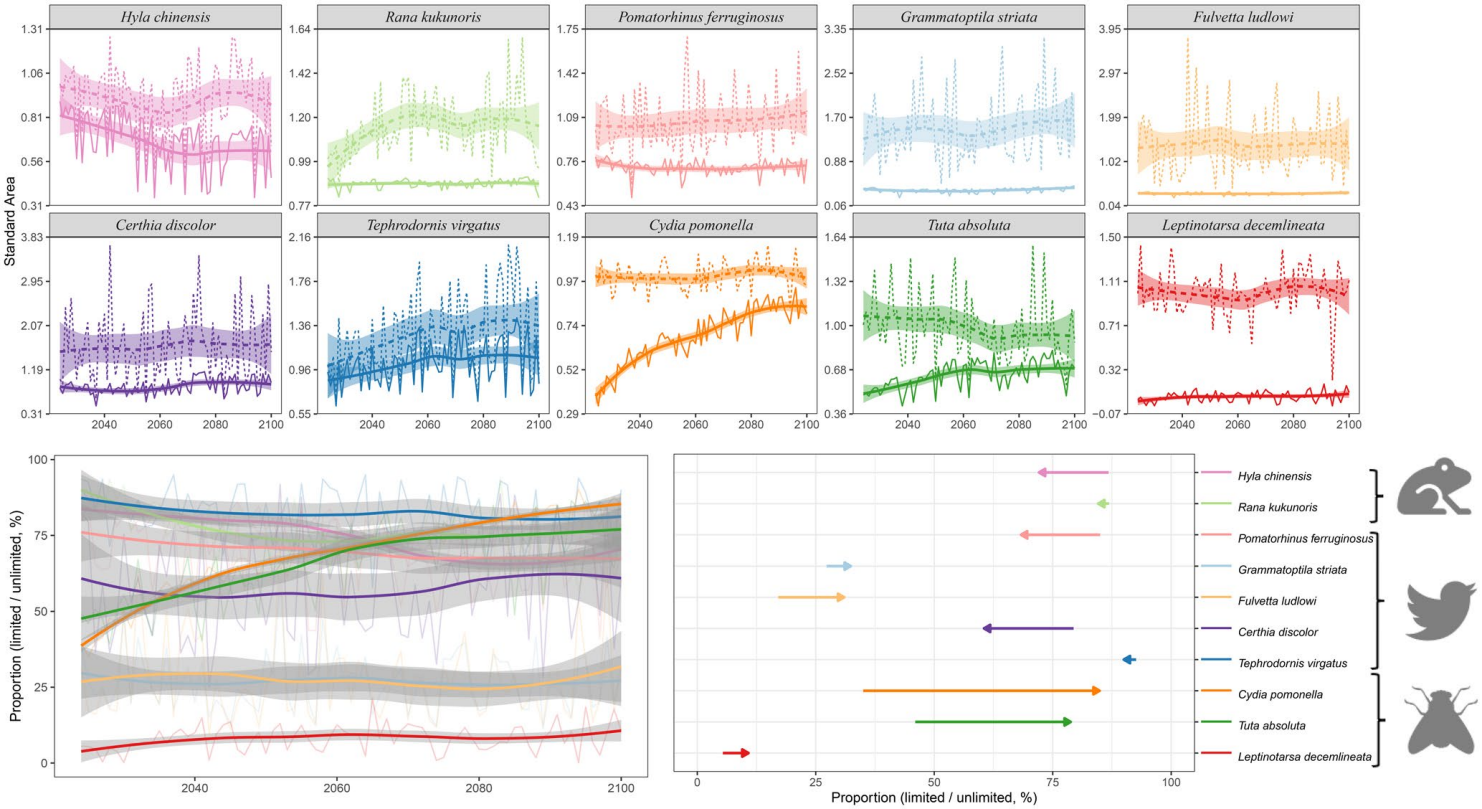
Projected changes under different dispersal scenarios from 2024 to 2100. The standardized suitable habitat areas under unlimited dispersal scenarios (dotted lines) and limited dispersal scenarios (solid lines) were projected with confidence interval in gray shade (a). The limited dispersal scenario incorporated species‐specific movement constraints. The proportion stands for the percentage of the suitable habitat area under the limited dispersal scenario relative to the unlimited dispersal scenario (b, c). In panel (b), each colored line corresponds to a different species, with thin pale lines representing annual values and thick lines with confidence intervals showing smoothed trends over time. In panel (c), the length of each line represents the proportional change from the present (2024) to the future (2100), and the direction of the arrow indicates whether the change is an increase or a decrease.

Without considering dispersal, the potential distribution area of seven species increased with climate change, while three species experienced a decrease in their potential distribution area under future climatic scenarios (Figure 2a). Among the species, five patterns emerged: (1) The potential distribution area increased linearly, represented by *P. ferruginosus* and *C. discolor* . (2) The potential distribution area followed a pattern of increase–decrease–increase and fluctuates greatly between years, represented by *R. kukunoris* , *G. striata* , *F. ludlowi* , and *T. virgatus* . (3) The potential distribution area followed a decreasing–increasing–decreasing trend, represented by *H. chinensis* and *L. decemlineata* . (4) The potential distribution area showed no significant change, as represented by *C. pomonella*. (5) The species with a decreasing potential distribution area is *T. absoluta*. Compared to the scenario where dispersal is considered, without considering dispersal, the potential distribution area of species shows greater fluctuations in the future (Figure 2).

When considering dispersal, four species showed a significant increase in potential distribution area with climate change: *T. virgatus* , *C. pomonella*, *T. absoluta*, and *L. decemlineata* . A declining trend is observed in the potential distribution areas of two species, namely *H. chinensis* and *P. ferruginosus* . The other four species showed little change in potential distribution area in the dispersal scenario (Figure 2a, Figure S3). Compared to the scenario without dispersal limitations, when dispersal is considered, the change in the potential distribution area of species in the future follows a continuous curve with minimal fluctuations.

### 3.3 Relative Change in Species' Potential Distribution Area With and Without Dispersal Limitation Under Climate Change

Under the climate change scenario with dispersal limitation, the relative change in potential distribution area was classified into three categories (Figure 2b,c): (1) Decreased Proportion: 40% of species ( *H. chinensis* , *R. kukunoris* , *P. ferruginosus*, and *T. virgatus* ) showed a noticeable decrease in the proportion of potential distribution area when considering dispersal limitation. This indicates that the difference between the scenarios with and without dispersal becomes more significant with climate change. (2) Unchanged Proportion: 30% of species ( *G. striata* , *F. ludlowi*, and *C. discolor* ) showed little change in the proportion of their potential distribution area after considering dispersal limitation. (3) Increased Proportion: 30% of species showed an increase in the proportion of their potential distribution area with dispersal limitation. This group includes the three invasive insects with strong dispersal abilities (*C. pomonella*, *T. absoluta*, and *L. decemlineata* ; Figure S3).

### 3.4 Changes in Species' Potential Distribution Under Different Climate Scenarios

Under the RCP8.5 climate scenario (high emissions), the potential distribution area of 10 species decreased compared to the RCP4.5 scenario (moderate emissions). This decline suggests that harsher climatic conditions, such as rising temperatures and changes in precipitation patterns, may restrict the suitable habitats of these species. Furthermore, the remaining suitable areas became more fragmented (Figure S4a,e,f,j). As shown in Figure S4, these changes highlight the potential adverse impacts of more severe climate change scenarios on species distribution and ecosystem stability (Figure S4).

## 4 Discussion

Under climate change, the potential suitable habitats of species may shift. Case 1 represents an optimistic but often unrealistic upper bound, where dispersal imposes no limitation and species can fully track shifting climatic niches. While commonly used to estimate the maximum potential future range, this assumption may overestimate species' ability to persist in rapidly changing environments. Case 2 represents a highly conservative scenario in which dispersal is entirely constrained. This assumption highlights the potential severity of range loss if species are unable to colonize newly suitable areas, providing a lower bound for persistence and emphasizing the importance of dispersal corridors. Considering realistic dispersal abilities, species can be further divided into those with strong or weak dispersal capacity. Cases 3 and 4 reflect more ecologically realistic situations in which dispersal ability varies among species. Species with strong dispersal capacity (Case 3) can partially or largely track the shifting climate envelope, thereby buffering the impacts of climate change and maintaining relatively stable distributions through time. In contrast, species with weak dispersal capacity (Case 4) are likely to experience a growing mismatch between their current distribution and newly emerging suitable habitats. This mismatch can lead to progressive range contraction, increased population isolation, and a heightened risk of extinction. These scenarios illustrate the critical role of dispersal ability in determining species' responses to climate‐driven habitat changes, highlighting that both the rate of habitat shift and the species' dispersal capacity jointly influence persistence and range dynamics.

Our results demonstrate that the consideration of species‐specific dispersal ability influences projections of future suitable habitats under alternative climate change scenarios. For species with strong dispersal abilities like *C. pomonella* and *T. absoluta* (Figure 2), they exhibited minimal differences in projected future suitable habitats between models that considered dispersal constraints (Figure 2) and those that relate to Case 1 and Case 3 (Figure 1b,g). This suggests that such species can effectively track shifting climatic conditions, thereby maintaining population stability. However, for species with limited dispersal capabilities, they showed substantial discrepancies between models considering dispersal or not. When dispersal constraints were incorporated, the predicted future habitat areas were markedly reduced in *R. kukunoris* and *H. chinensis* (Figure 2), which can correspond to Case 2 and Case 4 (Figure 1c,h). For strong dispersers, such as many bird and wind‐dispersed plants, it is rapid to move to and colonize new habitats. This ability enables them to adapt to changing climatic conditions more effectively, therefore reducing extinction risk. For weak dispersers like certain amphibians and mammals, they'll face challenges in relocating to newly suitable habitats and are more susceptible to population declines or extinctions as their habitats change. In addition, with climate change, their potential suitable habitats may become more fragmented, indicating a potential loss of habitat connectivity. Such fragmentation can hinder species dispersal, reduce genetic exchange between populations, and increase the risk of local extinctions. The range shifts may not be entirely prevented but rather significantly delayed and may require multiple generations or external assistance to behave in response to climate. This pattern aligns with the empirical studies showing that species with greater dispersal abilities have successfully expanded their ranges in response to climate change, whereas species with limited mobility face greater climate‐driven range contraction (Schloss et al. 2012).

These findings presented above again further underscore the critical importance of incorporating species‐specific dispersal capabilities into SDMs. Neglecting to account for their abilities to move to their suitable habitat can result in overly optimistic assessments of their extinction risk under climate change scenarios. For species with intrinsically limited dispersal ability, even though they have sufficiently large potential ranges that could theoretically support their population, they may still be unable to track the shifting climatic conditions. For some species whose migration is constrained by extrinsic dispersal barriers, the availability of future suitable habitat does not necessarily convert into increased survival probability. Even if climatically favorable areas emerge, these species may still be unable to cross such barriers. This situation is particularly relevant to species surviving on the edge of habitat, such as montane species, island species, etc. (Hollenbeck and Sax 2024, Johnston et al. 2012, Vazačová and Münzbergová 2014), whose movements are inherently constrained by geography. The combination of intrinsic and extrinsic limitations introduces significant uncertainty in assessing species' extinction risks and conservation priorities.

While neglecting dispersal limitations can lead to underestimation of extinction risk in conservation, it may conversely result in overestimation of invasion risk in preventing biological invasions. This situation can lead to misallocation of prevention resources and unnecessary costs. Although certain species, especially plants and insects, are identified as highly invasive species, their risk of invasion cannot merely be based on their climatic suitability. Not all invasive species possess strong dispersal abilities; thus, assessing invasion risks requires consideration of species‐specific traits (Zhang et al. 2023). Dispersal ability remains a key determinant of potential distribution, even for invasive plants (Lázaro‐Lobo et al. 2025). A study relevant to invasive insect Spongy moth ( *Lymantria dispar* ) also points out that its actual distribution may be constrained by various factors, leading to a slower spread rate than predicted (Alalouni et al. 2013; Camerini 2009; Faske et al. 2019). The widespread invasive freshwater clam *Corbicula fluminea* in North America also has been shown to be restricted by unsuitable climate conditions rather than habitat itself (McDowell et al. 2014). The barrier should also be included as an important factor when concerning invasive species' range expansion. As is the case with *C. ponemella* (Figure 2, Figure S3h), which is a severe agricultural pest in China, it demonstrates that despite models indicating nearly the entire country as a suitable distribution range, it fails to establish in the Qinghai‐Tibet Plateau and southeast China due to dispersal barriers and limitations. Previous studies indicate that topographic distance and isolation strongly constrain the vertical migration of plants within the same mountain range (Peyre et al. 2020). In aquatic ecosystems, physical barriers such as watershed divisions, salinity gradients, and current patterns significantly restrict the movement of invasive species. For instance, many freshwater invaders, despite their broad climatic tolerance, cannot disperse across disconnected water systems (Jones et al. 2021), which cannot be neglected when modeling their distribution. Accurately assessing invasion risks necessitates the integration of both intrinsic species traits and extrinsic environmental barriers into predictive models to ensure effective conservation planning and management strategies.

Given the inherent unpredictability of human activities, this study did not include human‐mediated dispersal into analyses. However, such processes remain a key area of concern and represent an important direction for future research. Understanding how anthropogenic factors influence both native species conservation and invasive species spread will be essential for developing comprehensive strategies under global climate change.

Considering dispersal limitations in SDM applications is essential for improving the accuracy of biodiversity predictions under climate change. By comparing predictions with and without dispersal constraints, we demonstrate that ignoring dispersal can lead to overestimations of future suitable habitat for weak dispersers, underestimating their extinction risk, while for strong dispersers, the differences between models with and without dispersal constraints remain minimal. This highlights the need for species‐specific movement data to ensure SDMs provide realistic assessments of species' ability to track shifting climatic conditions. As SDMs are increasingly playing an important role in species conservation and habitat management, integrating dispersal processes will be crucial in ensuring effective habitat protection, translocation efforts, and invasion risk assessments.

## Author Contributions

**Mengge Duan:** formal analysis (equal), visualization (equal), writing – original draft (equal). **Jiahua Xing:** data curation (equal), investigation (equal), visualization (equal), writing – original draft (equal). **Xiudeng Zheng:** conceptualization (equal), writing – review and editing (equal). **Chaodong Zhu:** conceptualization (equal), writing – review and editing (equal). **Huijie Qiao:** conceptualization (equal), funding acquisition (equal), project administration (equal), supervision (equal), validation (equal), writing – review and editing (equal).

## Funding

This work was supported by the National Key Research and Development Program of China (2025YFC2609100) and the National Natural Science Foundation of China (32271732).

## Conflicts of Interest

The authors declare no conflicts of interest.

## Supporting information


**Figure S1:** Species occurrence data for 10 species.
**Figure S2:** Interannual variation trends of environmental variables in the study area. The blue line represents the RCP8.5 climate scenario, while the pink line represents the RCP4.5 climate scenario.
**Figure S3:** Habitat suitability indices and potential distribution in future under the RCP4.5 scenario. Each facet represents a species: Hyla chinensis (a), Rana kukunoris (b), Pomatorhinus ferruginosus (c), Grammatoptila striata (d), Fulvetta ludlowi (e), Certhia discolor (f), Tephrodornis virgatus (g), Cydia pomonella (h), Tuta absoluta (i), Leptinotarsa decemlineata (j).
**Figure S4:** Potential distribution of species under future environmental conditions. The species represented by each panel can be found in Figure S3.
**Table S1:** Information for dispersal Ability of species.
**Table S2:** Information for species' model.

## Data Availability

The datasets underlying this article were derived from sources in the public domain. The data is public via https://doi.org/10.5281/zenodo.18976151. The link above is an anonymous link for editors and reviewers for peer review. Please copy the entire anonymous link, including the token to your browser. The formal DOI will be updated before the article is proofed.
