# Supplementary material for: Enhancing Species Distribution Models by Considering Dispersal Ability
Source: Ecol Evol. 2026 Apr 13;16(4):e73425. doi: 10.1002/ece3.73425 (PMC13071756; doi:10.1002/ece3.73425)
Supplement: Supplementary file 1 — Figure S1: Species occurrence data for 10 species. Figure S2: Interannual variation trends of environmental variables in the study area. The blue line represents the RCP8.5 climate scenario, while the pink line represents the RCP4.5 climate scenario. Figure S3: Habitat suitability indices and potential distribution in future under the RCP4.5 scenario. Each facet represents a species: Hyla chinensis (a), Rana kukunoris (b), Pomatorhinus ferruginosus (c), Grammatoptila striata (d), Fulvetta ludlowi (e), Certhia discolor (f), Tephrodornis virgatus (g), Cydia pomonella (h), Tuta absoluta (i), Leptinotarsa decemlineata (j). Figure S4: Potential distribution of species under future environmental conditions. The species represented by each panel can be found in Figure S3. Table S1: Information for dispersal Ability of species. Table S2: Information for species' model. [file ECE3-16-e73425-s001.docx]

**Supplementary materials**


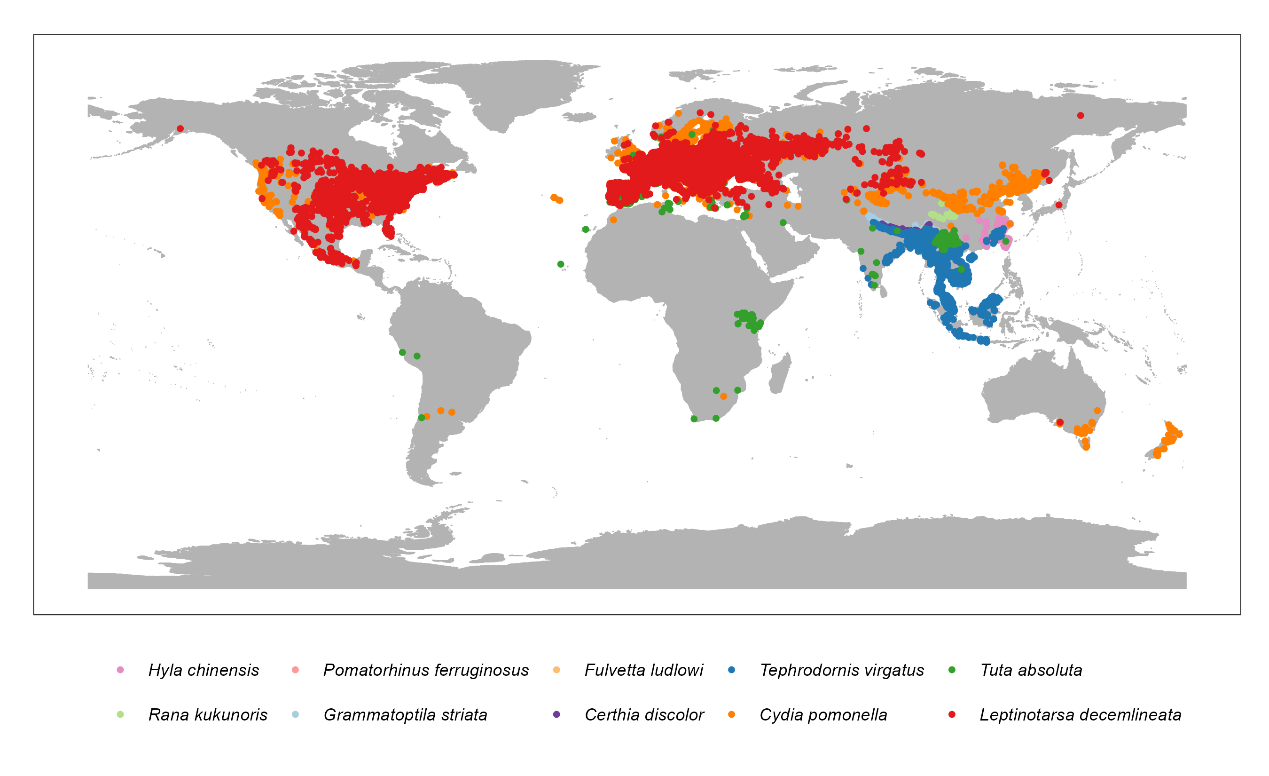


**Figure S1 Species occurrence data for ten species.**


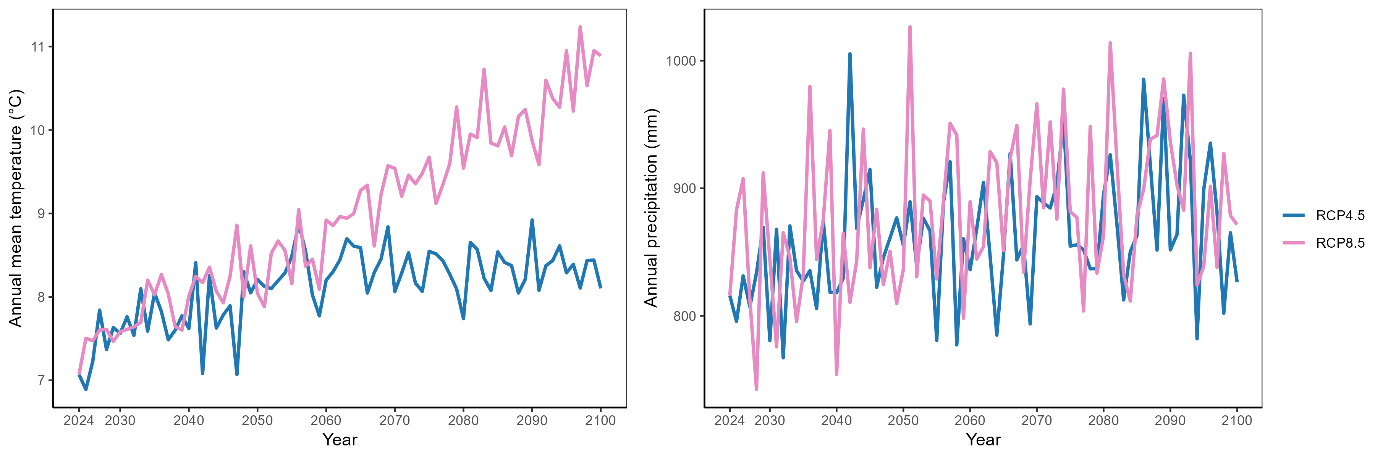


**Figure S2** **Interannual variation trends of environmental variables in the study area.** The blue line represents the RCP8.5 climate scenario, while the pink line represents the RCP4.5 climate scenario.


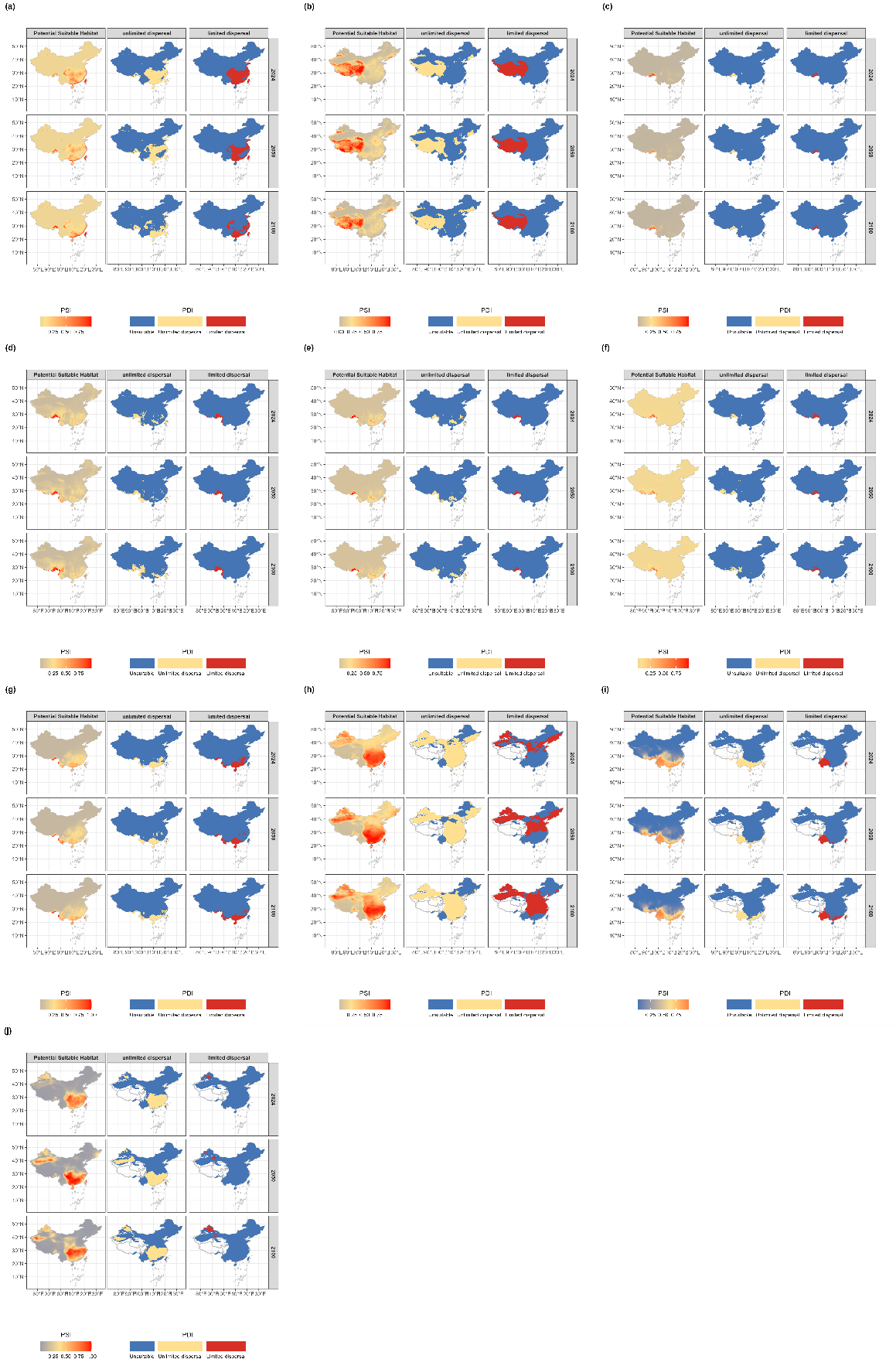


**Figure S3** **Habitat suitability indices and potential distribution in future under the RCP4.5 scenario.** Each facet represents a species: *Hyla chinensis* (**a**)*,* *Rana kukunoris* (**b**)*, Pomatorhinus ferruginosus* (**c**)*,* *Grammatoptila striata* (**d**)*, Fulvetta ludlowi* (**e**)*, Certhia discolor* (**f**)*,* *Tephrodornis virgatus* (**g**)*, Cydia pomonella* (**h**)*, Tuta absoluta* (**i**)*,* *Leptinotarsa decemlineata* (**j**)*.*


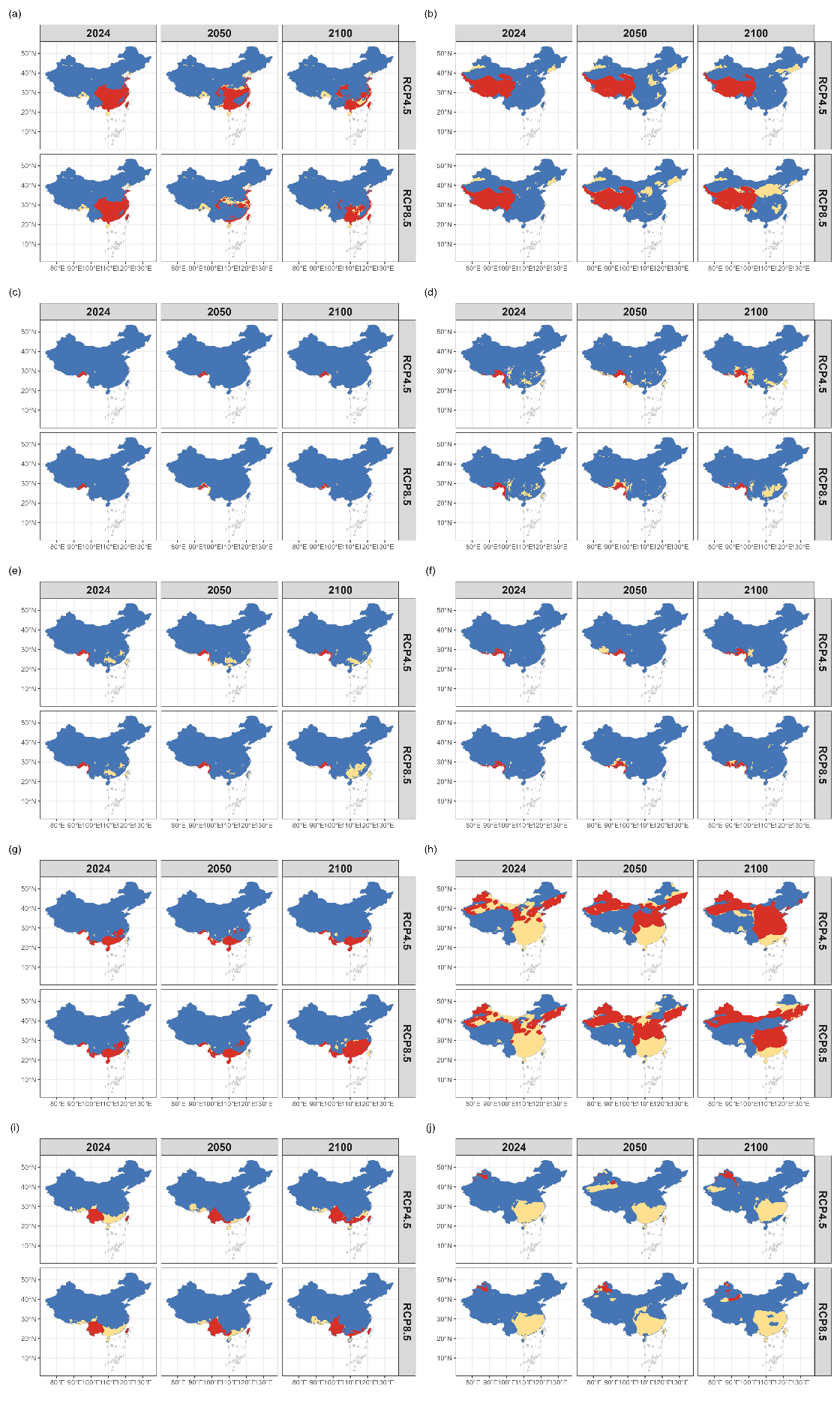


**Figure S4** **Potential distribution of species under future environmental conditions.** The species represented by each panel can be found in Fig. S3.

**Table S1** Information for dispersal Ability of species.

| Species | Classification | Type | Max dispersal distance(km)/year |  |
| --- | --- | --- | --- | --- |
| *Hyla chinensis* | amphibians | native | 1.00 (estimate) |  |
| *Rana kukunoris* | amphibians | native | 1.00 (estimate) |  |
| *Pomatorhinus ferruginosus* | bird | native | 0.72 (calculate) |  |
| *Grammatoptila striata* | bird | native | 1.65 (calculate) |  |
| *Fulvetta ludlowi* | | bird | native | 4.44 (calculate) |
| *Certhia discolor* | bird | native | 6.48 (calculate) |  |
| *Tephrodornis virgatus* | bird | native | 13.9 (calculate) |  |
| *Cydia pomonella* | insect | invasive | 10.00 (estimate) |  |
| *Tuta absoluta* | insect | invasive | 20.00 (estimate) |  |
| *Leptinotarsa decemlineata* | insect | invasive | 50.00 (estimate) |  |

| Species | Sample size | Climate variables | Model Evaluation | | |
| --- | --- | --- | --- | --- | --- |
|  |  |  | Partial AUC | TSS | Kappa |
| *Hyla chinensis* | 205 | BIO01, BIO02, BIO12, BIO15 | 1.83 | 0.81 | 0.75 |
| *Rana kukunoris* | 21 | BIO01, BIO03, BIO04, BIO12 | 1.73 | 0.83 | 0.99 |
| *Pomatorhinus ferruginosus* | 122 | BIO01, BIO02, BIO12, BIO19 | 1.95 | 0.90 | 0.55 |
| *Grammatoptila striata* | 646 | BIO01, BIO12, BIO14 | 1.82 | 0.79 | 0.47 |
| *Fulvetta ludlowi* | 59 | BIO03, BIO12, BIO19 | 1.98 | 0.87 | 0.73 |
| *Certhia discolor* | 202 | BIO01, BIO02, BIO12, BIO15 | 1.97 | 0.95 | 0.48 |
| *Tephrodornis virgatus* | 1416 | BIO01, BIO02, BIO12, BIO19 | 1.66 | 0.82 | 0.84 |
| *Cydia pomonella* | 5309 | BIO01, BIO03, BIO12, BIO15 | 1.59 | 0.64 | 0.77 |
| *Tuta absoluta* | 575 | BIO01, BIO04, BIO12, BIO17 | 1.67 | 0.72 | 0.91 |
| *Leptinotarsa decemlineata* | 9068 | BIO01, BIO03, BIO12, BIO15 | 1.72 | 0.74 | 0.64 |

**Table S2** Information for species’ model.
